# Design and Synthesis of Lactose, Galactose and Cholic Acid Related Dual Conjugated Chitosan Derivatives as Potential Anti Liver Cancer Drug Carriers

**DOI:** 10.3390/polym13172939

**Published:** 2021-08-31

**Authors:** Yili Ding, Wutong Cui, Chamakura V. N. S. Vara Prasad, Bingyun Wang

**Affiliations:** 1 Life Science Department, Foshan University, Foshan 528000, China; linlincui123456@163.com (W.C.); bywang63@163.com (B.W.); 2 Das Pharma, Turangi P.O., Kakinada 533016, India; vchamakura@gmail.com

**Keywords:** lactose, galactose, cholic acid, dual conjugated chitosan derivatives, sorafenib, solubility

## Abstract

Cholic acid and galactose or lactose dual conjugated chitosan derivatives were designed and synthesized as potential anti liver cancer drug carriers, their structures were characterized through proton NMR spectra, elemental analysis, size distribution, zeta potential, and scanning electron microscope image studies. The ability of the dual conjugates to enhance the aqueous solubility of the cancer drug sorafenib was evaluated. The entrapment efficiency (EE%) and drug content (DC%) of sorafenib in the inclusion complexes were measured. The chitosan dual conjugate with cholic acid and galactose was found to be best in enhancing the aqueous solubility of sorafenib. The solubility of sorafenib in water has increased from 1.7 µg/mL to 1900 µg/mL which is equal to 1117-fold increase in its solubility due to the inclusion complex with chitosan conjugate.

## 1. Introduction

Many of the existing cancer drugs with low water-solubility, non-specific targeting of cancer cells, and lower stability face a barrier for the drugs to reach the tumor area with maximum efficacy. The design and synthesis of the drug carriers with the cancer-specific tumor biomarkers and other functional groups to increase cancer drug’s tumor-targeting ability, absorptive transportation, in vivo stability, and systemic solubility, while reducing the adverse effects, is a potential therapeutic approach to treat cancer diseases with the high efficacy.

It was shown that the conjugation of carriers with antibodies and their variable fragments, peptides, nucleic aptamers, vitamins, and carbohydrates can lead the drug delivery to cancer cells [1]. The targeted drug delivery system is comprised of three components: a therapeutic agent, a targeting moiety, and a carrier system [2]. 

Chitosan, a natural polysaccharide [3], has received increasing attention due to its outstanding physical and biological properties such as biocompatibility, biodegradability, nontoxicity, low-immunogenicity, abundant availability, unique mucoadhesivity, and inherent pharmacological properties [4,5,6]. The chemical modification of chitosan through conjugating with hydrophobic moiety imparts amphiphilicity, which enables it in the formation of self-assembled nanoparticles [7], and the resulting hydrophobic cores of nanoparticles could act as reservoirs or microcontainers for various bioactive substances. Cholic acid, an endogenous amphiphilic steroid, is used as the shuttle to deliver drugs precisely to the liver [8,9,10,11,12,13,14,15]. The cholic acid conjugated chitosan nanoparticles were demonstrated as the drug carriers for paclitaxel. The paclitaxel-loaded nanoparticles showed remarkably high anticancer activity compared to that of paclitaxel in Cremophor EL-ethanol formulation against B16F10 cells [16]. Carbohydrate-bearing chitosans were also found to be promising liver-targeted [17,18] nanocarriers for hepatic drug targeted delivery [19,20,21,22]. 

**Figure 1:**
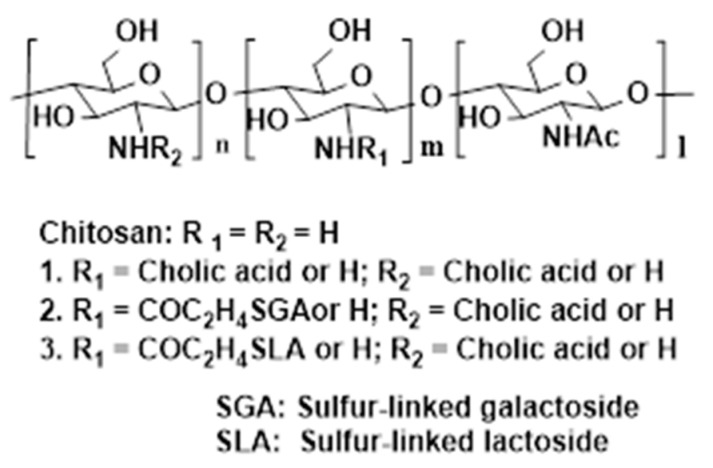
The structures of designed compounds **1**, **2** and **3**.

Thus, the ability of mono-conjugated chitosan derivatives, such as cholic acid conjugated chitosan, lactose linked chitosan, and galactose conjugated chitosan as drug carriers to deliver the anticancer agents to the liver has been known for some time. As part of our interest in employing biomolecules as drug carriers for dual targeting [23] of liver cancer cells, we anticipate that the bifunctionalized chitosan compounds can also be employed as drug carriers for targeting liver cancer cells, and these chitosan-linked molecules may increase the drug’s targeting efficacy, apart from improving drug’s water solubility and stability in comparison with monofunctionalized chitosan conjugates. In this communication, we report preliminary results on the design and synthesis of dual functionalized chitosan derivatives, and their evaluation in increasing the liver cancer drug’s water solubility. The proposed galactose or lactose and cholic acid dual-linked chitosan compounds may act as the polysaccharide nanosized drug carriers, and their structures (**1**, **2** and **3**) are listed in Figure 1. 

Sorafenib is an orally administered multi-kinase inhibitor that prevents tumor growth by anti-angiogenic, anti-proliferative, and/or pro-apoptotic effects [24], and is practically insoluble in water (1.7 μg/mL) [25]. Currently, sorafenib is one of the earliest approved drugs in the market for treating liver cancer. However, its non-specific uptake may lead to serious adverse events such as the development of second primary cancer, difficulty breathing, pleural effusion [26], and may result in less efficacy [27]. A commonly used technique to increase the water solubility of drugs is by supramolecular complexation [28]. The results of employing the above chitosan conjugates **1**, **2**, and **3** as the dual liver cell targeting nano-drug carriers, to increase the water solubility of sorafenib [29], will be described as part of a demonstration of the usefulness of the described chitosan conjugates.

## 2. The Experiments

### 2.1. Materials and Instruments 

Chitosan oligosaccharide (25 KDa, 90% deacetylated products), cholic acid, 3-dicyclohexyl carbodiimide (DCC), *N*-hydroxy succinimide (NHS), lactose, galactose, and sorafenib were purchased from Sigma. Other solvents and chemicals were used in the reagent grade. ^1^H NMR spectra were recorded by Bruker spectrometer operating at 400 MHz using D_2_O as lock solvent, and the sample concentration was maintained at 1.0 mg/mL. The elemental analysis was carried out in Elementar (Langenselbold, Hesse Germany, Vario EL cube) instrument and the scanning electron microscope images were recorded on a Micro-surface morphology (Hitachi, Tokyo, Japan). The dynamic light scattering experiment was carried out on an electrophoretic LS spectrophotometer (ELS-8000) and the UV spectra were obtained using a Lambda 25 UV-Vis spectrophotometer (PerkinElmer, Waltham, MA, USA). 

### 2.2. Experimental Procedures

***N*-Hydroxysuccinimidoyl cholic ester** [17] (**4**): A mixture of cholic acid (0.32 g, 0.8 mmol), 3-dicyclohexyl carbodiimide (DCC) (0.175 g, 0.8 mmol), and *N*-hydroxy succinimide (NHS) (0.095 g, 0.8 mmol) in THF (5 mL) was stirred at room temperature for 24 h under nitrogen in the presence of DIEA (0.1 mL). The precipitated product dicyclohexylurea (DCU) was filtered off, and the solution of NHS ester **4** was used directly for the next reaction.

***N*-Hydroxysuccinimidoyl 3-(*β*-D-galactopyranosylthio)-propionate** (**6**): A mixture of 3-(*β*-D-galactopyranosylthio)-propionic acid [30] (**5**) (0.22 g, 0.8 mmol), 3-dicyclohexyl carbodiimide (DCC) (0.175 g, 0.8 mmol), and *N*-hydroxy succinimide (NHS) (0.095 g, 0.8 mmol) in THF/DMF (1:1, 5 mL) was stirred at room temperature for 24 h under nitrogen in the presence of DIEA (0.1 mL). The precipitated product dicyclohexylurea (DCU) was filtered off, and the solution of NHS ester **6** was used directly for the next reaction.

***N*-Hydroxysuccinimidoyl 3-(*β*-D-lactopyranosylthio)-propionate** (**8**): A mixture of 3-(*β*-D-lactopyranosylthio)-propionic acid (**7**) [31] (0.34 g, 0.8 mmol), 3-dicyclohexyl carbodiimide (DCC) (0.175 g, 0.8 mmol), and *N*-hydroxy succinimide (NHS) (0.095 g, 0.8 mmol) in THF/DMF (1:1, 5 mL) was stirred at room temperature for 24 h under nitrogen in the presence of DIEA (0.1 mL). The precipitated product dicyclohexylurea (DCU) was filtered off, and the solution of NHS ester **8** was used directly for the next reaction.

**Cholic acid conjugated chitosan** (**1**): To the solution of chitosan (0.1 g) in a mixture of DMSO/H_2_O (10:1, 10 mL) was added compound **4** obtained from 0.2 g of cholic acid in THF. The reaction mixture was stirred for 48 h at room temperature. Then, the resulting solution was precipitated by adding an excess amount of acetone/methanol (2:1). The precipitates were recovered by centrifugation and dried under a vacuum after washing out with acetone. The solid was dissolved into distilled water, and the solution was dialyzed against deionized water in 3000 Da dialysis bags for 72 h. The resulting solution was filtered through the 1.2 μm pore sized syringe filter to remove large aggregates. Finally, the cholic acid conjugated chitosan (**1**) was obtained by lyophilization as a solid. Elemental analysis: C, 39.15%; N, 6.03%.

**Cholic acid and 3-(*β*-D-galactopyranosylthio)-propionic acid conjugated chitosan** (**2**): To the solution of chitosan (0.1 g) in a mixture of DMSO/H_2_O (10:1, 10 mL) was added compound **4** (obtained from 0.2 g of cholic acid) and compound **6** obtained from 0.15 g of 3-(*β*-D-galactopyranosylthio)-propionic acid (**5**) in THF. The reaction mixture was stirred for 48 h at room temperature under nitrogen. The resulting solution was precipitated by adding an excess amount of acetone/methanol (2:1). The precipitate was recovered by centrifugation and dried under a vacuum after washing out with acetone. The solid was dissolved into distilled water, and the solution was dialyzed against deionized water in 3000 Da dialysis bags for 72 h. The resulting solution was filtered through the 1.2 μm pore-sized syringe filter to remove large aggregates. Finally, the cholic acid and 3-(*β*-D-galactopyranosylthio)-propionic acid conjugated chitosan (**2**) was obtained by lyophilization as a white solid. Elemental analysis: C, 38.05%; N, 5.67%; S, 3.42%.

**Cholic acid and 3-(*β*-D-lactopyranosylthio)-propionic acid conjugated chitosan** (**3**): To the solution of chitosan (0.1 g) in a mixture of DMSO/H_2_O (10:1, 10 mL) was added compound **4** (obtained from 0.2 g of cholic acid) and compound **8** obtained from 0.3 g of 3-(*β*-D-lactopyranosylthio)-propionic acid (**7**) in THF. The reaction mixture was stirred for 48 h at room temperature, then the resulting solution was precipitated by adding an excess amount of acetone/methanol (2:1). The precipitate was recovered by centrifugation and dried under a vacuum after washing out with acetone. After dissolving in distilled water, the solution was then dialyzed against deionized water in 3000 Da dialysis bags for 72 h. After dialysis, the resulting solution was filtered through the 1.2 μm pore-sized syringe filter to remove large aggregates. Finally, the cholic acid and 3-(*β*-D-lactopyranosylthio)-propionic acid conjugated chitosan (**3**) was obtained by lyophilization as a white solid. Elemental analysis: C, 38.29%; N, 6.23%; S, 1.66%.

**Substitution degrees:** The substitution degree of cholic acid DS in conjugate **1**, substitution degrees of cholic acid (DS) and galactosyl part (DSgal) in conjugate **2**, and substitution degrees of cholic acid (DS) and lactosyl part (DSlac) in conjugate **3** were obtained based on their elemental analysis data and following methods:

For cholic acid conjugated chitosan derivative **1**, since the starting material chitosan contains 10% of *N*-acetyl derivatives, every acetyl group contains two carbon atoms, every monosaccharide contains one nitrogen atom and six carbon atoms, and each cholic acid fragment contains 24 carbon atoms. Then, for every 100 monosaccharides, the ratio of C/N should be:

C/N = [(100 × 6 × 12) + (10 × 2 × 12) + (24 × 12 × DS)]/(100 × 1 × 14) (1)

Based on the ratio of C/N from elemental analysis, we can obtain the degree of substitution of cholic acid DS in conjugate **1**.

For galactose and cholic acid conjugated chitosan derivative **2**, every monosaccharide contains one nitrogen atom, each galactosyl fragment contains one sulfur atom, for every 100 monosaccharides, the ratio of S/N should be:

S/N = (1 × 32 × DS_gal_)/(100 × 1 × 14) (2)

Based on the ratio of S/N from elemental analysis, we are able to know the degree of substitution of galactosyl fragment (DS_gal_) in conjugate **2**. Each cholic acid fragment contains 24 atoms, each galactosyl fragment contains nine carbon atoms, for every 100 monosaccharides, the ratio of C/N should be:

C/N = [(100 × 6 × 12) + (10 × 2 × 12) + (9 × 12 × DSgal) + (24 × 12 × DS)]/(100 × 1 × 14)(3)

Based on the ratio of C/N from the elemental analysis, we are able to find the degree of substitution of cholic acid (DS) in conjugate **2**.

By using a similar method, we can find the degrees of substitution of lactosyl fragment and cholic acid in conjugate **3**.

**Preparation of self-aggregated nanoparticles and the size distribution of nanoparticles and zeta potential determination**: Chitosan conjugate **1**, **2** or **3** was dispersed in water (1.0 mg/mL), and the mixture was shaken gently at room temperature for 48 h. After three times sonication (2 min for each time at 80 W), the solution was filtered through a filter (1.2 μm) to remove any particulate matter. The size distribution of nanoparticles and zeta potential was determined by dynamic light scattering (DLS). The DLS experiment was carried out using an electrophoretic LS spectrophotometer (ELS-8000) equipped with a He-Ne laser operating at 632.8 nm at 25 °C and a fixed scattering angle of 90°.

**Scanning electron microscope images of chitosan conjugates**: To observe the morphology of self-aggregated nanoparticles of chitosan conjugates **1**, **2** and **3**, the sample solutions (1 mg/mL) were dropped onto the carbon-coated 300 mesh copper grids. Then, the grids were air-dried and imaged using a scanning electron microscope (HITACHI, Tokyo, Japan) at an accelerating voltage of 80 kV.

**Preparation of sorafenib loaded nanoparticles with chitosan conjugates 1, 2 and 3**: The solution of sorafenib (5 mg) in methanol (1 mL) was added into the solution of chitosan conjugate **1**, **2** or **3** (10 mg) in water (10 mL) slowly with stirring at room temperature. After ultrasound (80 W) for 30 min, the mixture was put into water (200 mL) for dialysis for 24 h. The solution was then centrifuged for 20 min at the rate of 10,000–12,000 rpm, and the resulting supernatant aqueous solution was lyophilized to give the sorafenib complexes as **S1** (10.5 mg), **S2** (12.3 mg), and **S3** (11.8 mg).

**Determination of drug content and encapsulating efficiency of sorafenib-loaded nanoparticles:** The solution of sorafenib in acetonitrile was measured for its UV absorbance, and its maximum absorption is at 251 nm. It is interesting to note that the chitosan conjugates **1**, **2** and **3** have no UV absorption at 251 nm. Based on the UV absorption of sorafenib in acetonitrile at 251 nm at different concentrations, a linear regression equation between the amount of sorafenib and UV absorption was obtained as Y = 1.6682 X + 0.0451, where the Y represented the amount of sorafenib, and the X represented the UV absorbance. The sorafenib-loaded nanoparticles (5 mg) were dissolved in distilled water in 2 mL, sonicated for 1 min (using a probe-type sonicator at 80 W), and the UV absorbance of the resulting solution was measured; 1.6 mg was found in sample **S1**, 1.9 mg was found in sample **S2**, and 1.7 mg was found in sample **S3**. Based on these data, the drug contents (DC%) and encapsulating efficiency (EE%) were calculated by using the following formulas:

DC% = (weight of the drug in complex/weight of feeding polymer and drug) × 100%

EE% = (weight of the drug in complex/weight of the feeding drug) × 100%

## 3. Results and Discussion

**Scheme 1:**
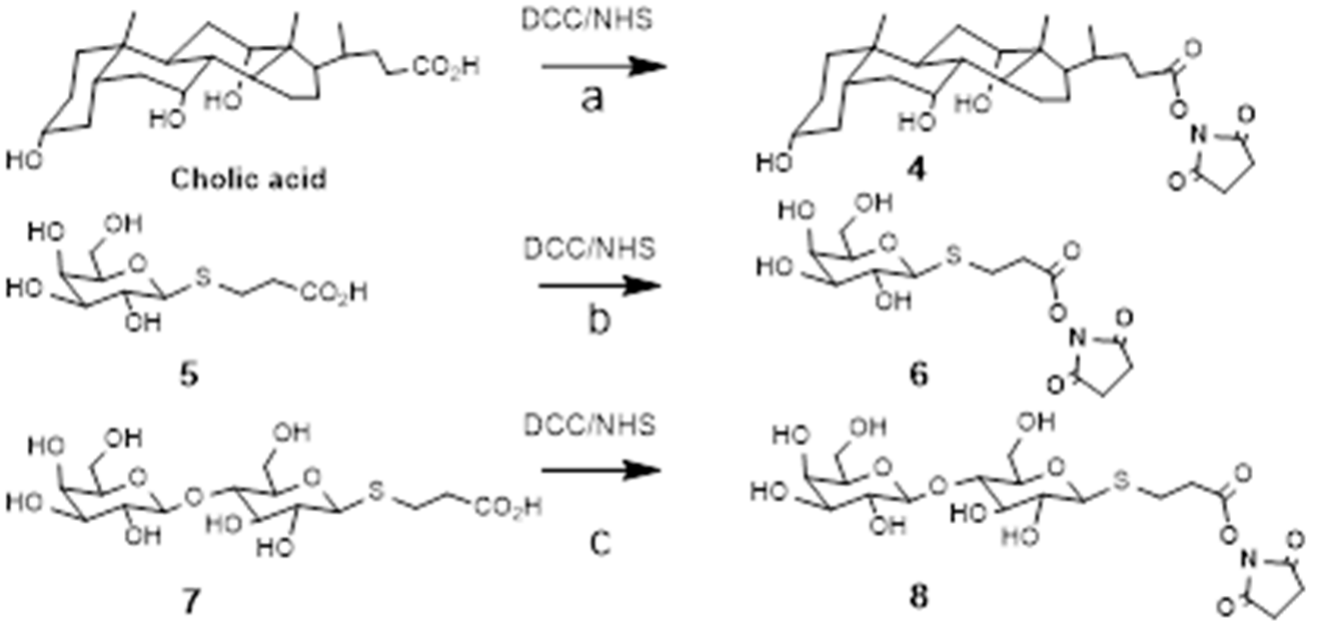
Reagents and conditions. (**a**): cholic acid (0.8 mmol), DCC (0.8 mmol), NHS (0.8 mmol), dry THF (5 mL), DIEA (0.1 mL), room temperature, N_2_, 24 h; (**b**): compound **5** (0.8 mmol), DCC (0.8 mmol), NHS (0.8 mmol), dry THF/DMF (4:1, 5 mL), DIEA (0.1 mL), room temperature, N_2_, 24 h; (**c**): compound **7** (0.8 mmol), DCC (0.8 mmol), NHS (0.8 mmol), dry THF/DMF (4:1, 5 mL), DIEA (0.1 mL), room temperature, N_2_, 24 h.

The amino groups in chitosan were linked to cholic acid, galactose and lactose related acid derivatives (**4**, **6** and **8**) through amide bond formation to generate compounds **1**, **2** and **3** respectively. Galactose and lactose related acid derivatives **5** and **7** were prepared by using the literature-reported procedures [30,31]. Thereafter, cholic acid, compounds **5** and **7** were activated by reaction with DCC and NHS in dry THF or DMF/THF to convert the carboxylic acid groups to NHS ester compounds **4**, **6** and **8** (Scheme 1) respectively.

**Scheme 2:**
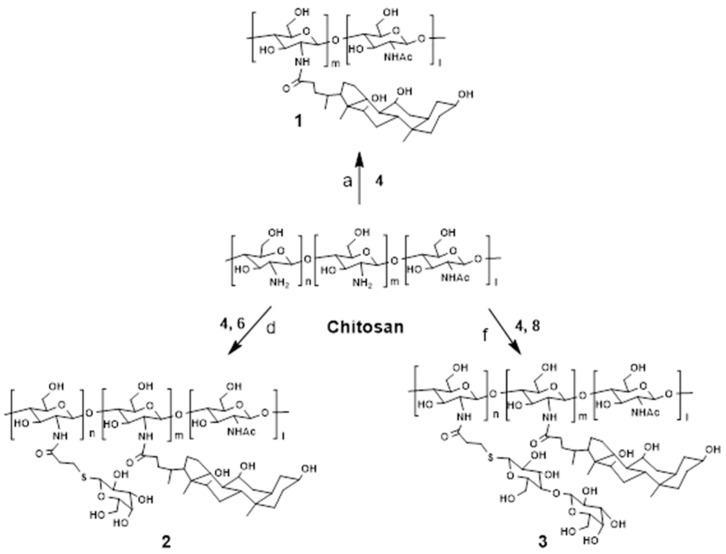
Reagents and conditions. (**a**): chitosan (100 mg), **4** (from 200 mg of cholic acid), dry DMSO (5 mL), room temperature, under nitrogen, 48 h; (**b**): chitosan (100 mg), **4** (from 200 mg of cholic acid), **6** (from 150 mg of compound **5**), dry DMSO (5 mL), room temperature, under nitrogen, 48 h; (**c**): chitosan (100 mg), **4** (from 200 mg of cholic acid), **8** (from 300 mg of compound **7**), dry DMSO (5 mL), room temperature, under nitrogen, 48 h.

The NHS esters **4**, **6** and **8** were then reacted with chitosan in DMSO for 48 h and subjected to dialysis in water for 48 h. Upon lyophilization of the resulting solutions, the cholic acid conjugated chitosan derivative **1**, cholic acid and galactose conjugated chitosan derivative **2**, cholic acid and lactose conjugated chitosan derivative **3** were obtained as white solids (Scheme 2).

The chitosan conjugates **1**, **2** and **3** are confirmed by their proton NMR spectra. The degree of substitution of cholic acid (DS) in compound **1**, the degrees of substitution of cholic acid (DS) and galactosyl fragment (DSgal) in compound **2**, the degrees of substitution of cholic acid (DS) and lactosyl fragment (DSlac) in compound **3** were obtained based on their elemental analysis. Further, these conjugates were analyzed for their size distribution and zeta potentials of their aqueous solutions. The morphologies of the above conjugates were observed using scanning electron microscopy.

The proton NMR spectra of chitosan, cholic acid conjugated chitosan derivative **1**, cholic acid and galactose conjugated chitosan derivative **2**, cholic acid and lactose conjugated chitosan derivative **3** were recorded in D_2_O (1 mg/1 mL). By comparison of the spectra of conjugates **1**, **2** and **3** with that of chitosan, the peaks from 0.6 ppm to 1.7 ppm in the spectra of compounds **1**, **2** and **3** indicated the cholic acid fragments were linked to chitosan. Although the proton signals in the proton NMR of the conjugates **1**, **2** and **3** confirmed the presence of cholic acid moiety, the degree of substitution of the cholic acid, galactose and lactose moieties could not be accurately calculated by comparing the ratio of the cholic acid proton to sugar protons due to the self-aggregation of conjugates in the aqueous phase.

The substitution degree of cholic acid in conjugate **1**, the substitution degrees of galactose and cholic acid in conjugate **2**, and the substitution degrees of lactose and cholic acid in conjugate **3** were calculated according to their sulfur and the nitrogen contents, which were determined by elemental analysis (Table 1). For conjugate **1**, from the ratio of C and N contents, the percentage of cholic acid substitution was determined to be 5.72%; while for conjugate **2**, from the ratio of S and N contents, the percentage of galactosyl substitution degree was found to be 26.38%, in addition, the ratio of C and N contents indicated the cholic acid substitution percentage as 6.68%; and finally for conjugate **3**, from the ratio of S and N contents, the percentage of lactosyl substitution degree was found to be 11.65%, and further from the ratio of C and N contents, the cholic acid percentage substitution was found to be 3.97% for this conjugate. The pattern of the degree of substitution in both mono-conjugate and the dual conjugates is in the expected range of the literature values to the close analogs of cholic acid, galactose and lactose. For example, the degree of substitution in cholic acid mono-conjugate **1** is in line with the reported values in the literature to its close analog [32]. Meanwhile, the substitution degrees of dual-conjugates (**2** and **3**) correspond to less than the mono-conjugates of carbohydrate derivatives of chitosans reported in the literature [33]. As expected, this may be due to competition and steric hindrance between the substitution groups in the dual-conjugates. The degree of substitution of galactosyl fragment in the dual-conjugate **2** is higher than that of the lactosyl fragment in dual-conjugate **3**. This may be expected, as the smaller size monosaccharide galactosyl group in compound **2** may have less steric hindrance than the larger lactosyl group in compound **3**.

**Figure 2:**
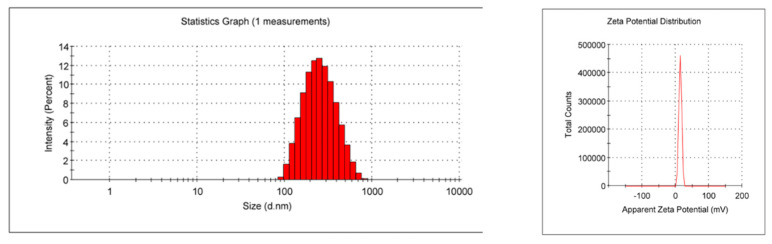
Size distribution and Zeta potential of chitosan Conjugate 1.

**Figure 3:**
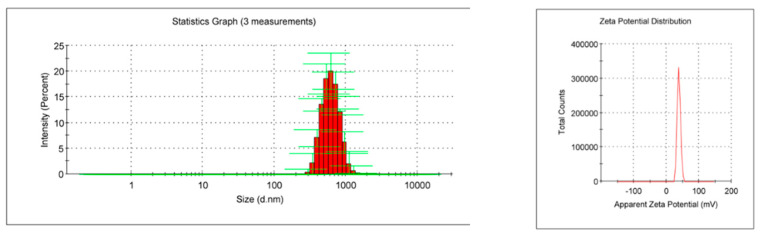
Size distribution and Zeta potential of chitosan Conjugate **2**.

**Figure 4:**
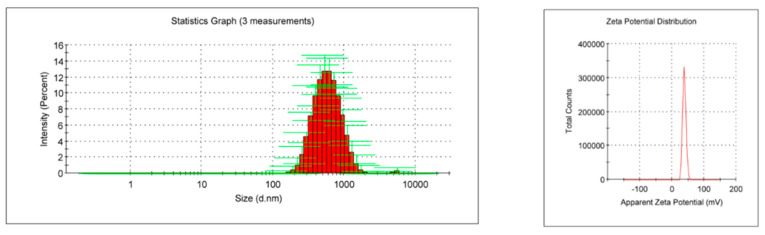
Size distribution and Zeta potential of chitosan Conjugate **3**.

The size distributions (Table 2) of nanoparticles of compounds **1**, **2** and **3** were determined by dynamic light scattering and the results complement elemental analysis data. Accordingly, the size distribution of mono-conjugate **1** is in agreement with cholic acid-related conjugate reported in the literature [32]. As expected, the sizes (nM) of NPs of dual-conjugates **2** and **3** are higher than that of mono-conjugate **1** (see Figure 2, Figure 3 and Figure 4). The zeta potentials of self-aggregated nanoparticles of chitosan conjugates **1**, **2**, and **3** were listed in Table 2. The zeta potential of dual conjugates **2** and **3** (Figure 3 and Figure 4) are higher than that of monoconjugate **1** (Figure 2), and this corresponds to the size distribution of conjugates. The observed zeta potentials pattern is counter-intuitive, as due to the higher degree of substitution in chitosan-conjugates, it is expected that the derivatives will show less surface charge due to less availability of charged species. However, the observed zeta potentials pattern may be reasoned, due to the pattern of formation of nanoparticles and their surface area. The larger surface area of the dual-conjugates may contribute to their larger zeta potentials in comparison to that of the monoconjugate.

**Figure 5:**
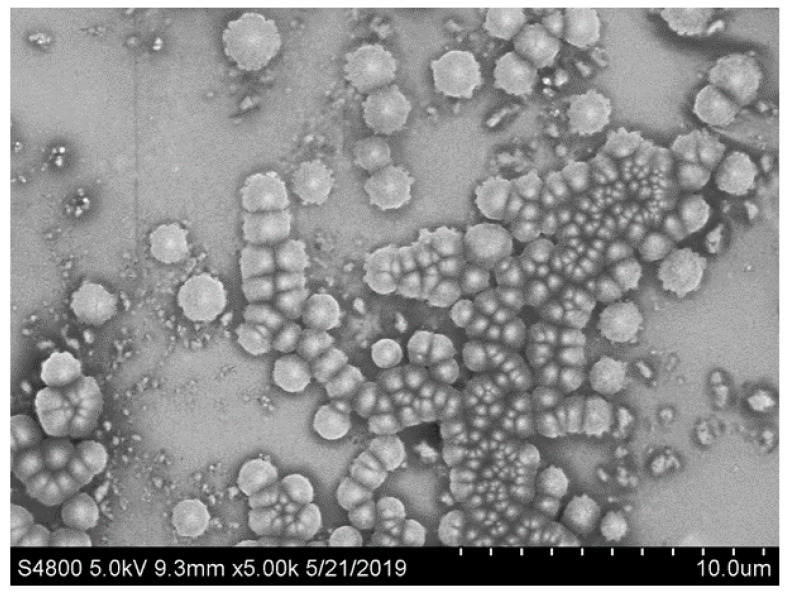
SEM image of cholic conjugated chitosan derivative **1**.

**Figure 6:**
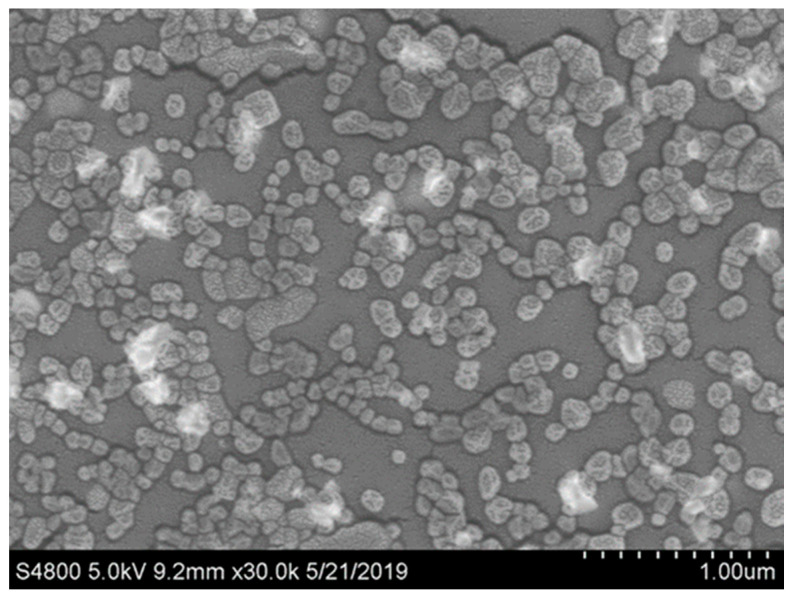
SEM image of cholic conjugated chitosan derivative **2**.

**Figure 7:**
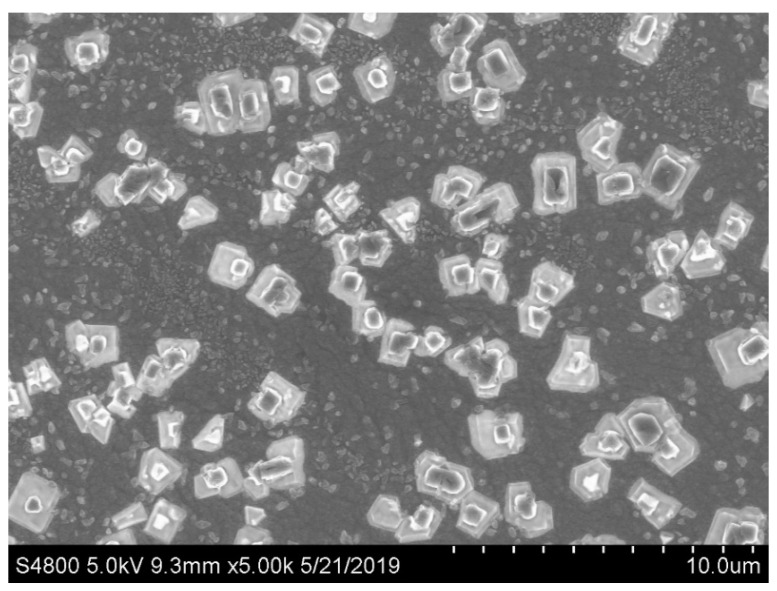
SEM image of cholic conjugated chitosan derivative **3**.

Morphologies of self-aggregated nanoparticles of chitosan conjugates **1**, **2** and **3** were observed by scanning electron microscope, and the SEM photographs of chitosan conjugates **1**, **2** and **3** were shown in Figure 5, Figure 6 and Figure 7. The monoconjugate nanoparticles shape appears to be distinctly different from that of dual-conjugates.

**Figure 8:**
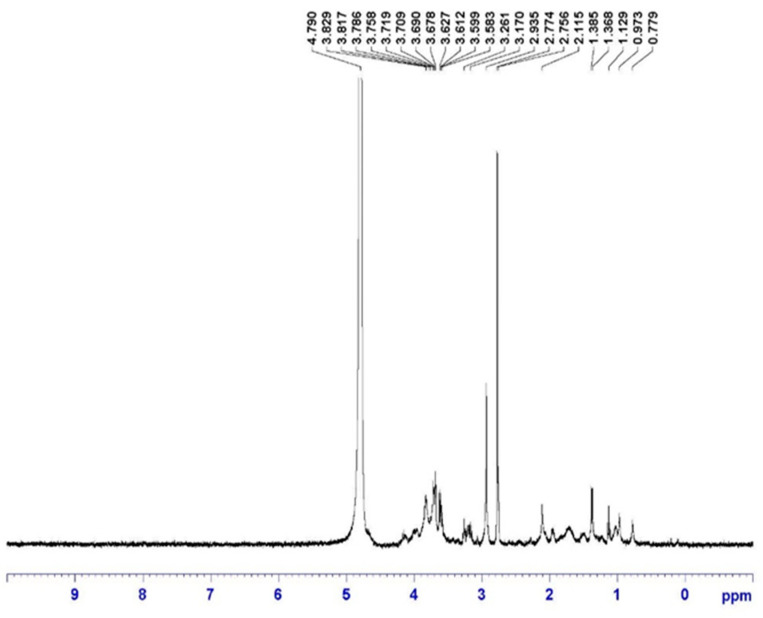
Proton NMR spectrum of cholic acid conjugated chitosan derivative **1** in D_2_O.

**Figure 9:**
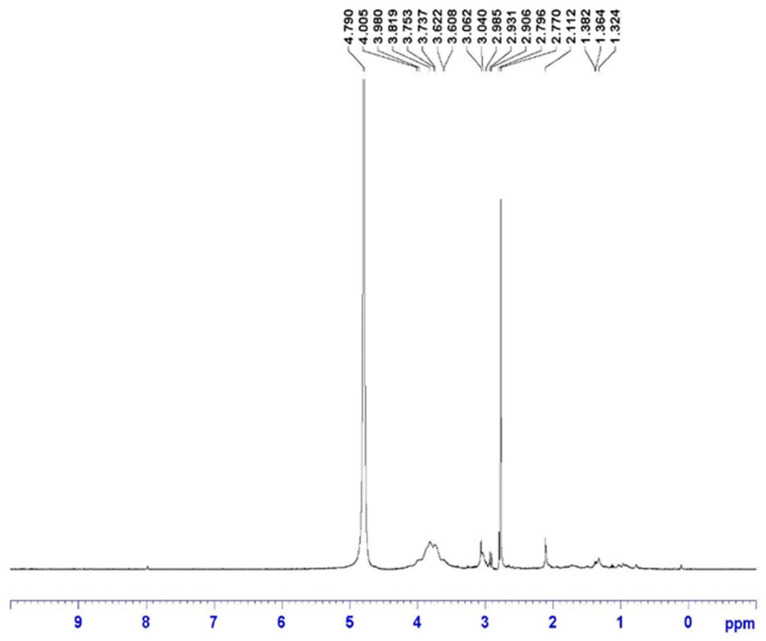
Proton NMR spectrum of galactose and cholic acid conjugated derivative **2** in D_2_O.

**Figure 10:**
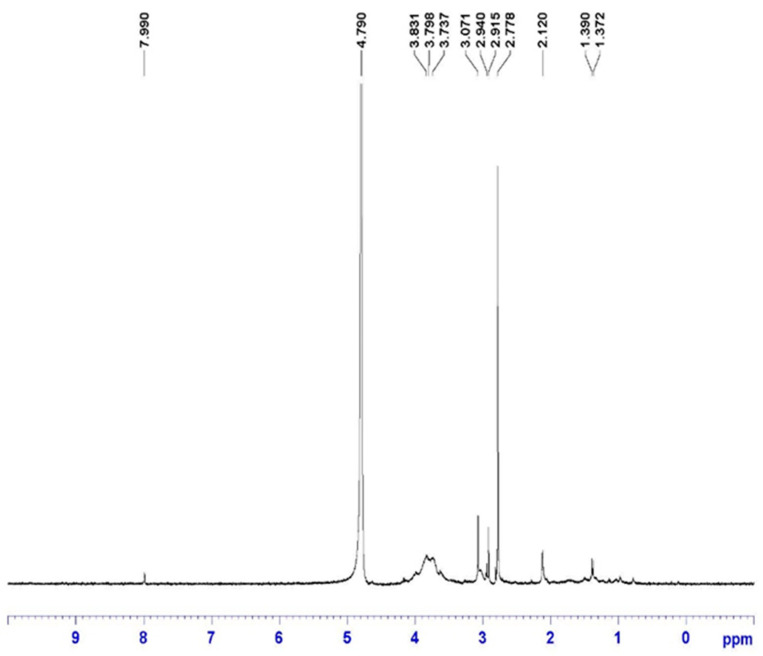
Proton NMR spectrum of lactose and cholic acid conjugated chitosan derivative **3** in D_2_O.

**Figure 11:**
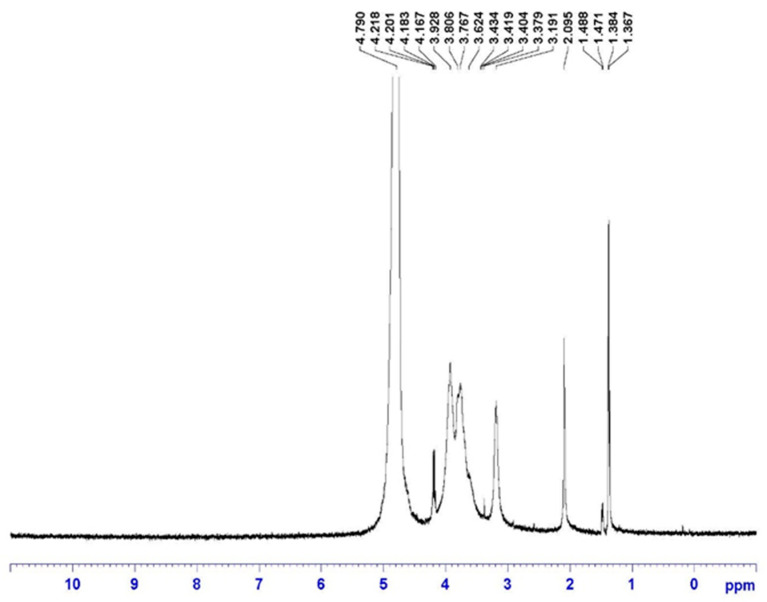
Proton NMR spectrum of chitosan in D_2_O.

The proton nmr spectra of the conjugates **1**, **2**, and **3** were recorded and shown in Figure 8, Figure 9 and Figure 10. By comparison with the proton nmr spectrum of chitosan (Figure 11), it was confirmed that these three conjugates contain the cholic fragments, as there are peaks from 2.0 ppm to 0.8 ppm in their nmr spectra. 

Chitosan conjugates **1**, **2,** and **3** as the dual liver cell targeting nano-drug carriers may increase sorafenib’s water solubility. The sorafenib inclusion compounds **1S**, **2S,** and **3S** were obtained as white solids.

The entrapment efficiency (EE%) and drug content (DC%) of sorafenib in the inclusion complexes **1S**, **2S** and **3S** were measured based on the absorption of UV light in an ultraviolet spectrophotometer against the standard curve obtained.

Thus, for the sorafenib inclusion complex **1S**, the amount of sorafenib was found to be 1.6 mg. Accordingly, the calculated values of DC% (weight of the drug in micelles/weight of feeding polymer and drug) and the EE% (weight of the drug in micelles/weight of the feeding drug) were found to be 32% and 67%, respectively. For the sorafenib inclusion complex **2S**, the amount of sorafenib was calculated to be 1.9 mg. The DC and EE values for this complex were found to be 38% and 93%, respectively. Lastly, for the sorafenib inclusion complex **3S**, the amount of sorafenib was found to be 1.7 mg. The DC% and the EE% values for complex **3S** were found to be 34% and 80%, respectively.

As we hypothesized, the sorafenib chitosan conjugate inclusion compounds **1S, 2S**, and **3S** were found to have increased the solubility of the drug. Most importantly, inclusion complex **2S** demonstrated about an 1117-fold increase in the solubility of sorafenib in comparison to its solubility without the aid of chitosan conjugate.

## 4. Conclusions

In this preliminary study, we reported the design and synthesis of the chitosan conjugate of cholic acid, the dual conjugate of cholic acid and galactose, and dual conjugate of cholic acid and lactose, and evaluated their ability to improve the aqueous solubility of cancer drug sorafenib. The evaluated conjugates demonstrated their ability to enhance the aqueous solubility of sorafenib in comparison to that of sorafenib alone. Among the conjugates studied, dual chitosan conjugate with cholic acid and galactose (**2**) was found to be best in enhancing the aqueous solubility of sorafenib. Further, evaluation of dual conjugate **2** for improving the solubility of additional drugs and their biological studies is in progress, and will be reported in due course.

## Figures and Tables

**Table 1:** Degrees of substitution of cholic acid, galactosyl fragment and lactosyl fragment in chitosan conjugates.

Samples	Elemental Analysis (%)				Degree of Substitutions (%)	
	C	N	S	Cholic Acid	Galactosyl Moiety	Lactosyl Moiety
**1**	39.15	6.03		5.72		
**2**	38.05	5.67	3.42	6.68	26.38	
**3**	38.29	6.23	1.66	3.97		11.65

**Table 2:** Size distribution and Zeta potential of the self-aggregated nanoparticles of chitosan conjugates.

Samples	Critical Aggregation Concentration	Diameter (nm)	Zeta Potential (mV)
**1**	1 mg/mL	219.1	14.1
**2**	1 mg/mL	571.5	38.3
**3**	1 mg/mL	474.7	34.8

## Data Availability

Not applicable.
